# Study on a High-Boron-Content Stainless Steel Composite for Nuclear Radiation

**DOI:** 10.3390/ma14227004

**Published:** 2021-11-19

**Authors:** Wei-Qiang Sun, Guang Hu, Xiao-Hang Yu, Jian Shi, Hu Xu, Rong-Jun Wu, Chao He, Qiang Yi, Hua-Si Hu

**Affiliations:** 1 School of Nuclear Science and Technology, Xi’an Jiaotong University, Xi’an 710049, China; sunweiqiang@stu.xjtu.edu.cn (W.-Q.S.); xuhu@stu.xjtu.edu.cn (H.X.); 2 Department of Nuclear System Safety Engineering, Nagaoka University of Technology, Nagaoka 940-2188, Japan; xiangqi1219@sina.com; 3 State Key Laboratory of Light Alloy Foundry Technology for High-End Equipment Shenyang Research Institute of Foundry Co., Ltd., Shenyang 110022, China; shijian@chinasrif.com; 4 Wuhan Second Ship Design and Research Institute, Wuhan 430064, China; wurongjun@whhwtech.com; 5 Institute of Nuclear Physics and Chemistry, China Academy of Engineering Physics, Mianyang 621900, China; hechao@126.com (C.H.); yiq@163.com (Q.Y.)

**Keywords:** radiation-shielding material, optimization design, MCNP code, neutron, γ rays transmission experiment

## Abstract

In this research, a high-boron-content composite material with both neutron and γ rays shielding properties was developed by an optimized design and manufacture. It consists of 304 stainless steel as the matrix and spherical boron carbide (B_4_C) particles as the functional particles. The content of B_4_C is 24.68 wt%, and the particles’ radius is 1.53 mm. The density of the newly designed material is 5.17 g·cm^−3^, about 68.02% of that of traditional borated stainless steel containing 1.7 wt% boron, while its neutrons shielding performance is much better. Firstly, focusing on shielding properties and material density, the content and the size of B_4_C were optimized by the Genetic Algorithm (GA) program combined with the MCNP program. Then, some samples of the material were manufactured by the infiltration casting technique according to the optimized results. The actual density of the samples was 5.21 g cm^−3^. In addition, the neutron and γ rays shielding performance of the samples and borated stainless steel containing 1.7 wt% boron was tested by using an ^241^Am–Be neutron source and ^60^Co and ^137^Cs γ rays sources, respectively, and the results were compared. It can be concluded that the new designed material could be used as a material for nuclear power plants or spent-fuel storage and transportation containers with high requirements for mobility.

## 1. Introduction

In nuclear fuel reprocessing systems, borated stainless steel (BSS) is widely used in storage and transportation containers for spent nuclear fuel because of its excellent thermal neutron absorption performance and good mechanical properties [1,2,3]. The common BSS is based on 304 stainless steel with limited content of the boron element. At present, research mainly focuses on the influence of the boron content on steel’s mechanical and shielding properties [4,5,6,7]. Generally, as the boron content increases, the thermal neutron absorption capacity will increase but ductility, impact toughness, and corrosive resistance will decrease [8]. Because of the low solubility limits of the B element in borated stainless steel, excessive addition of boron will cause the formation of a fragile phase (Fe,Cr)_2_B making it difficult to prepare borated stainless steel [9,10]. The ASTM standard A887 covers eight types of BSS according to their content of B, and the weight percentage of boron is only 0.2–0.25 wt% [11]. For this reason, in this paper, spherical boron carbide (B_4_C) particles were added to a 304 stainless-steel matrix through the infiltration casting technique, which is different from the alloy casting method [12], to improve the boron content to nearly 20 wt%.

At the same time, the lighter the shield material, the better the mobility of spent-fuel storage and transportation containers. Therefore, it is of great significance to develop lightweight borated stainless steel. However, the shielding performance must be kept when the material density is lower. In fact, BSS shows a good neutrons shielding performance, because iron, carbon, and boron play their respective roles in shielding neutrons in different energy segments. For wide-range neutrons shielding, the following principles should be followed [13,14]. Firstly, fast neutrons are slowed down to medium energy by the inelastic scattering of heavy elements such as iron and tungsten, and then their energy is further slowed to a low energy region by elastic collision with neutrons of lighter elements such as hydrogen and carbon; then, the neutrons are completely absorbed through capture absorption by specific elements such as boron, lithium, and gadolinium. It is known that γ rays will be emitted in the process of inelastic collision and capture absorption; therefore, heavy elements such as iron and lead will be added to the neutron-shielding material. According to the above principles, it is possible to reduce the weight of BSS while keeping its neutron shielding performance unchanged by increasing the content of B and C and decreasing that of Fe. Considering the low solubility limits of boron in borated stainless steel [9] and the high boron content in B_4_C, if more B_4_C is added to the matrix material, the content of B can be increased. Because the density of B_4_C is 2.52 g cm^−3^, the higher its content is, the smaller the density of the composite is. This paper is based on this idea.

Firstly, combining the Monte Carlo Particle Transport Code-MCNP [15] with the Genetic Algorithm (GA) program and taking the neutron and γ rays shielding performance and material density as the optimization objectives, the content and the size of B_4_C carbide were optimized. The density of the designed material was 5.17 g cm^−3^, about 68.02% of that of BSS with 1.7 wt% boron, which is 304B6 type in the ASTM standard A887 and it is also a typical BSS used in the storage and transportation containers of spent nuclear fuel. Then, according to the design parameters, several material samples with the size of 10 cm × 2 cm × 10 cm were prepared by an already studied infiltration casting technique [12] and the true density of the samples was found to be 5.21 g cm^−3^. Lastly, the neutron and γ rays shielding performance of the material samples was tested using ^241^Am–Be neutron source and ^60^Co and ^137^Cs γ rays source devices. The results showed that, compared with BSS containing 1.7 wt% boron, the designed material showed a much better shielding performance for fast neutrons with an average energy of 4.5 MeV and 3.98 MeV, and its mass attenuation coefficient was equivalent to that of BSS for γ rays with an average energy of 0.662 MeV and 1.25 MeV. It is shown that this new designed material could be a candidate material for nuclear power plants or spent fuel storage and transportation containers with high requirements for mobility.

## 2. Materials and Methods

### 2.1. Theoretical Method

By interacting with matter, the intensity and energy of rays will change as they pass through a shield. For γ rays, they interact with electrons outside the nucleus, mainly through the photoelectric effect, Compton effect, and electron pair effect. Neutrons, because they are not charged, will interact with the nucleus, mainly through elastic scattering, inelastic scattering, and capture absorption. Simply speaking, the ray intensity will decay exponentially with the increase of the shield thickness [16,17], as follows

(1) $$N_{x}=N_{0}exp\left(-\mu \cdot x\right)=N_{0}exp\left(\frac{-\mu }{\rho }\cdot \rho \cdot x\right)=N_{0}exp\left(-\mu_{m}\cdot \rho \cdot x\right)$$

(2) $$N_{x}=N_{0}exp\left(-\Sigma \cdot x\right),\;\Sigma =10^{-24}\frac{N_{A}}{M}\cdot \rho \cdot \sigma$$

where x is the thickness of the shield material, μ and $\mu_{m}$ are linear attenuation coefficient and mass attenuation coefficient of the material to γ rays, respectively, σ and Σ are microscopic cross section and macroscopic cross section of the material to neutrons, respectively, $N_{A}$ is the Avogadro constant corresponding to 6.023 × 10^23^, M is the atomic weight, and ρ is the material density.

Equations (1) and (2) show that the radiation shielding performance of the material can be preliminarily judged by the linear attenuation coefficient and the neutron macroscopic cross section. The larger the linear attenuation coefficient and the macroscopic cross section, the better the shielding performance of the material. The mass attenuation coefficient and microscopic cross section of Fe, C, and B in different energy segments were obtained from databases [18,19] and were calculated according to Equations (1) and (2), as shown in Table 1 and Table 2.

For γ rays, the linear attenuation coefficients of iron are always higher than those of carbon and boron, from 1 × 10^−3^ MeV to 10 MeV. According to Equation (1), at the same thickness of the shield, the shielding performance of iron is much better than that of carbon and boron. However, for neutrons, the neutron macroscopic cross section of iron is not always higher than that of boron and carbon in different energy ranges. This means that for the fission spectrum with a wide energy spectrum, the neutron shielding performance of BSS material could be kept unchanged or even improve by increasing the content of carbon and boron and reducing the content of iron. Based on this theoretical analysis, a high-boron-content composite was optimized and prepared; the content of B was increased to nearly 20 wt% in this research.

### 2.2. Material Design

#### 2.2.1. Optimized Design Method

In order to improve the content of the B element and keep unchanged the neutrons and γ rays shielding performance of the material, while reducing its overall density, B_4_C was added to the 304 stainless steel matrix in the form of a sphere. The content and the size of boron carbide were optimized by the Genetic Algorithm (GA) program combined with the Monte Carlo N Particle Transport-MCNP program. The design progress can be regarded as a numerical optimization problem with constraints. The goal was to minimize the total dose of neutron and γ rays behind the shield and to minimize the material density. The shielding performance and the density were regarded as the optimization sub-objectives, and the total optimization objective was obtained by their weight addition. The matrix material, 304 stainless steel, was selected as a reference material for nondimensionalized sub-objectives, as follows,

(3) $$f{\left(X\right)}_{min}=a_{1}f_{H}\left(X\right)+a_{2}f_{\rho }\left(X\right),\;a_{1}+a_{2}=1$$

(4) $$f_{H}\left(X\right)=\frac{H_{t}}{H_{t}^{*}}=\frac{2.407H_{n}+7.77H_{g}}{2.407H_{n}^{*}+7.77H_{g}^{*}}$$

(5) $$f_{\rho }\left(X\right)=\frac{\rho_{x}}{\rho^{*}}$$

where $X=\left(x_{1},x_{2},x_{3}\right)$ is the variable vector including the mass fraction of B_4_C, the mass fraction of 304 stainless steel, and the radius of the B_4_C, with 0 <$\;x_{1}\;$< 0.25, 0 <$\;x_{2\;}$< 1.0, 1.0 mm <$\;x_{3\;}$< 2.5 mm; $f{\left(X\right)}_{min}\;$is the total optimization objective function; $f_{H}\left(X\right)$ is the material shielding performance sub-objective; $f_{\rho }\left(X\right)$ is the material density sub-objective; $a_{1}$ and $a_{2}$ are weight factors to adjust the weight of two sub-objectives; $H_{t}$ is the total dose equivalent of one fission behind the designed material (the average number released by one fission event were 2.407 neutrons and 7.77 γ photons); $H_{n}$ is the total dose equivalent of one fission neutron behind the designed material, including the secondary γ ray; $H_{g}$ is the total dose equivalent of one fission γ behind the designed material; $H_{t}^{*}$,$\;H_{n}^{*}$ and $H_{g}^{*}$ refer to the 304 stainless steel reference material; $\rho_{x}$ is the density of the designed material; $\rho^{*}$ is the density of 304 stainless steel, i.e., 7.9 g cm^−3^.

The Toolbox Function GA in MATLAB (R2016A) and Monte Carlo N Particle Transport (MCNP) program were used. There are seven main steps as follows:

(a) Calculate the total dose equivalent behind the 304 stainless steel material by MCNP, $H_{t}^{*}\;$(only once);

(b) Input the parameters of the variable vector in the GA program;

(c) Produce the ‘n’ file and ‘p’ file for simulating the neutron and γ rays transmission in the material by MATLAB;

(d) Calculate the input ‘n’ file and ‘p’ file by MCNP and produce the output ’no’ file and ‘po’ file;

(e) Extract the data showing the dose equivalent of neutrons in ’no’ file and γ rays in ‘po’ file and calculate the total dose equivalent;

(f) Calculate the fitness function value ($f{\left(X\right)}_{\min}$) by the Equations (3)–(5);

(g) The GA program will stop when the difference of fitness function value between two generations is less than 1 × 10^−6^ or the iteration times reaches the max generation number. If this does not work, new parameters of the variable vector will be produced, and then a new calculation will be performed.

#### 2.2.2. Shielding Property Calculation Model

**Figure 1:**
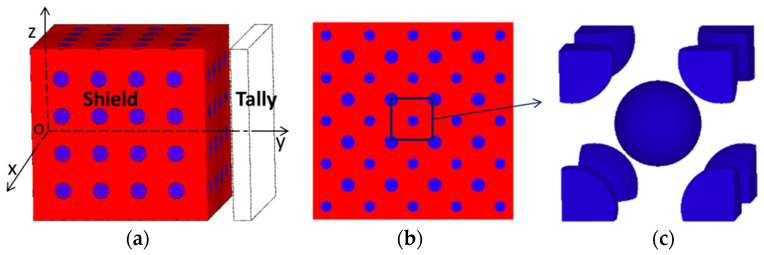
BCC Model for MCNP simulation; (**a**) the whole calculation model, (**b**) X-Z cross section of the model, (**c**) the BCC Model Unit.

The Uniform Filling Model, in which the elements are uniformly filling, is generally used for the calculation of radiation shielding. However, the size of B_4_C is relatively large, so the error will be very large when the Uniform Filling Model is also used. Therefore, the BCC Model (Body Center Cubic Model) was established to calculate the shielding property in MCNP, as shown in Figure 1. Different from the traditional Uniform Filling Model, B_4_C was packed in the shield in the form of spherical particles as body-centered cubic units rather than elements. The BCC Model Unit was first set up as the filling body and then was filled in the whole shield. The volume fraction of the B_4_C particle was realized by controlling the side length of the cubic unit. The unit side length was calculated according to the following formula

(6) $$l_{BCC}={\left(\left(\left(1+8\times \frac{1}{8}\right)\times \frac{4}{3}\pi r^{3}\right)/per\right)}^{\frac{1}{3}}$$

The radiation source was a uniform plane perpendicular to the y-axis and the size was 20 cm × 20 cm. The shield size was 20 cm × 20 cm × 20 cm. The F4 tally card in the MCNP program was used to calculate the flux behind the shield, and the flux was converted to the dose equivalent by multiplying by the Flux-to-Dose Factors.

### 2.3. Material Sample Manufacture

**Figure 2:**
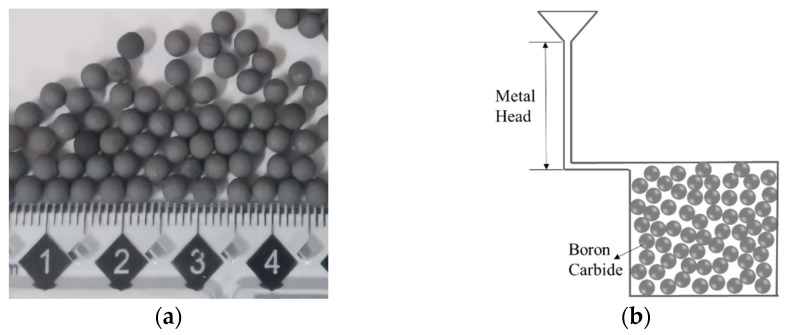
Boron carbide particles and casting process. (**a**) B_4_C particles with average 1.5 mm radius, (**b**) schematic diagram of infiltration casting.

According to the optimized B_4_C content (24.68 wt%) and its radius (1.53 mm), five material samples of 10 cm × 2 cm × 10 cm were prepared using the infiltration casting technique in a vacuum mid-frequency induction furnace [12]. In the preparation process, the matrix material was 304 stainless steel, and the average particle size of spherical B_4_C was 1.5 mm, as shown in Figure 2a. The B_4_C spheres were put into a ceramic mold and stacked up randomly and loosely, as shown in Figure 2b. During the preparation, the mold was pre-heated to 1000 °C, while the stainless steel was heated to 100–150 °C above its melting point. As the process was completed under vacuum, B_4_C would not commonly react with O_2_. The microstructure of the matrix material was still austenite at room temperature; no obvious phase transformation was detected. The true density of the prepared samples was 5.21 g·cm^−3^ as measured by the drainage method.

## 3. Neutron and γ Rays Transmission Measurements

The neutron and γ rays shielding properties of two kinds of material—the designed material and borated stainless steel with a boron content of 1.7 wt%—were tested on ^241^Am–Be neutron source and the ^60^Co and ^137^Cs γ rays source devices.

### 3.1. Neutron Transmission Measurement

#### 3.1.1. Neutron Source Parameter

**Figure 3:**
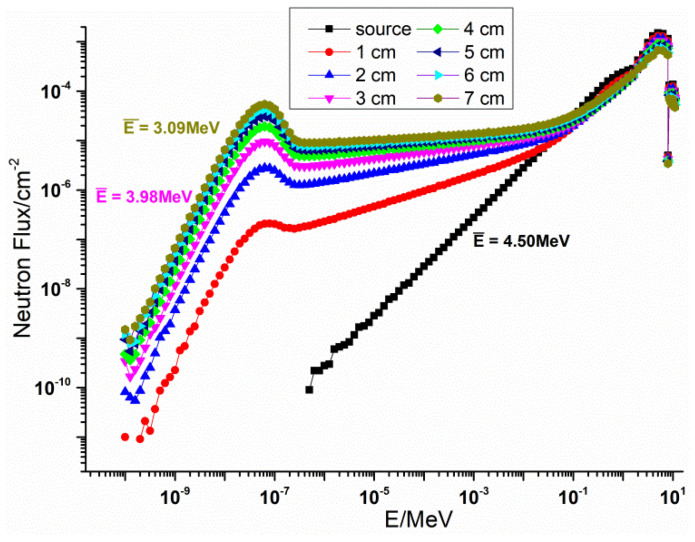
Energy spectrum and average energy of the ^241^Am–Be neutron source moderated by polyethylene layers with different thicknesses.

The ^241^Am–Be neutron source emis in the 4 π direction, showing an average energy of about 4.5 MeV and an activity of 5 Ci. In order to broaden the experiment energy point, polyethylene layers with different thicknesses were placed behind the ^241^Am–Be neutron source to slow down the neutrons and reduce the energy of the source spectrum. MCNP was used to simulate neutrons’ normalized energy spectrum after polyethylene moderation, as shown in Figure 3.

Because of the elastic scattering between polyethylene and neutrons, the energy spectrum of the source migrated. It can be seen in Figure 3 that, with the increase of the thickness of moderating polyethylene, the average energy and the proportion of high-energy neutrons decreased, while the proportion of medium- and low-energy neutrons increased. In the experiment, a non-polyethylene moderating layer and a 3 cm polyethylene moderating layer were applied. The average energy values were 4.5 MeV and 3.98 MeV, respectively.

#### 3.1.2. Neutron Experiment Layout

The neutron ambient dose equivalent (rate) meter is often used to measure the ambient dose equivalent produced by neutron radiation. Both neutrons that arrive directly from the source to the meter and neutrons scattered from the air, concrete walls, and other objects in the calibration room contribute to the measurement results [20]. A BH3105 type neutron ambient dose equivalent (rate) meter made in China was used to measure the dose equivalent behind the material samples in this experiment. When there are no moderating polyethylene or material samples, the measured value is directly proportional to the number of neutrons reaching the detector, since the conversion coefficient between the dose equivalent and the spectral average fluence can be regarded as a constant [21]. In fact, when the moderating polyethylene and material samples were set in the experiment, the energy spectrum of the neutron source changes; therefore, the conversion coefficient between dose equivalent and flux will also changed. However, considering that this experiment is a comparative experiment, it was assumed that the coefficient remained almost constant. The neutron transmittance of materials with different thickness can be calculated according to following formula

(7) $$T_{i}=\frac{\emptyset_{i}}{\emptyset_{0}}\approx \frac{D_{i}}{D_{0}},\;i=1,\;2,\;3,\;\ldots$$

where $T_{i}$ is the transmittance, $\emptyset_{0}$ is the ray flux caused by direct rays reaching the detector without sample material, $\emptyset_{i}$ is the ray flux behind the sample material caused by direct rays reaching the detector, $D_{0}$ is the dose equivalent caused by direct rays reaching the detector without the sample material, $D_{i}$ is the dose equivalent behind the sample material caused by direct rays reaching the detector.

**Figure 4:**
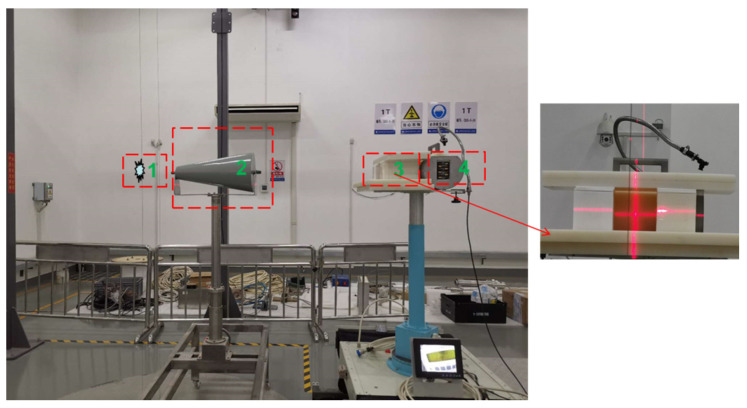
Layout of the neutron transmission experiment with a shadow cone and without polyethylene moderation (1: neutron source: lifted from the basement when in use; 2: shadow cone: shields the direct neutrons; 3: polyethylene shielding layer; 4: neutron ambient dose equivalent (rate) meters).

When there was no polyethylene moderation, a single experiment using a single sample material could be divided into four steps:

(a) The layout was Source Target + Detector: the total dose equivalent caused by neutrons reaching the detector position without material sample was measured;

(b) The layout was Source Target + Shadow Cone + detector: the shadow cone completely blocked the direct neutrons. At this time, the dose equivalent caused by the scattered neutrons was measured [20]. Combined with the results of step (a), the dose equivalent caused by direct neutrons reaching the detector without sample material could be obtained, $D_{0}$;

(c) The layout was Source Target + Material Sample + Detector: the total dose equivalent caused by neutrons reaching the detector in the presence of the material sample was measured;

(d) The layout was Source Target + Shadow Cone + Material Sample + Detector (as shown in Figure 4): combined with the results of step (c), the dose equivalent caused by direct neutrons reaching the detector position in the presence of the sample material could be obtained, $D_{i}$;

When we used a 3 cm polyethylene moderating layer, the experiment steps were basically the same as the above steps, except that the polyethylene moderating layer was added behind the source in each step.

### 3.2. Rays Transmission Measurement

#### 3.2.1. γ Rays Source Parameter

^60^Co and ^137^Cs γ ray sources are often used in γ rays shielding experiments. The parameters of the γ rays source devices used in this experiment are shown in Table 3.

#### 3.2.2. γ Rays Experiments Layout

During the γ rays experiments, a thermoluminescence personal dosimeter was used to measure the total cumulative dose behind the shielding material samples. The total cumulative dose was directly proportional to the number of γ rays reaching the detector. Therefore, the γ rays transmission of materials with different thickness could still be calculated according to Equation (7).

**Figure 5:**
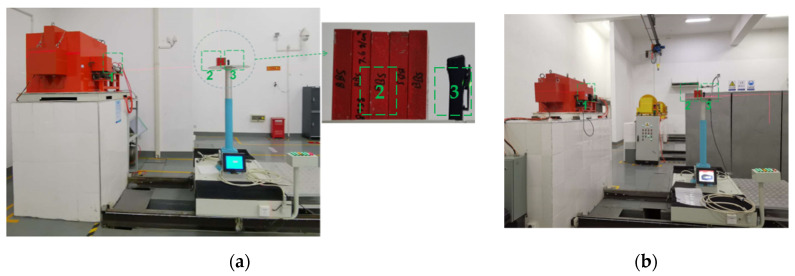
Layout of γ rays transmission experiments. (**a**) ^60^Co γ rays; (**b**) ^37^Cs γ rays. (1: γ rays outlet; 2: material samples; 3: thermoluminescence personal dosimeter).

The γ rays source transmission experiment was divided into two main steps:

(1) The layout was Source + Thermoluminescence personal dosimeter: the total cumulative dose reaching the detector without sample material was measured;

(2) The layout was Source + Material Sample + Thermoluminescence personal dosimeter: the total cumulative dose reaching the detector in the presence of the material sample was measured, as shown in Figure 5.

In order to ensure the accuracy of the data, each data point was measured 10 times, and its average value was taken. However, since we measured the cumulative dose, for each measurement, the cumulative dose was recorded every 30 s, that is, each data point was measured for 300 s.

## 4. Results and Discussion

### 4.1. Material Optimization Design Results

**Figure 6:**
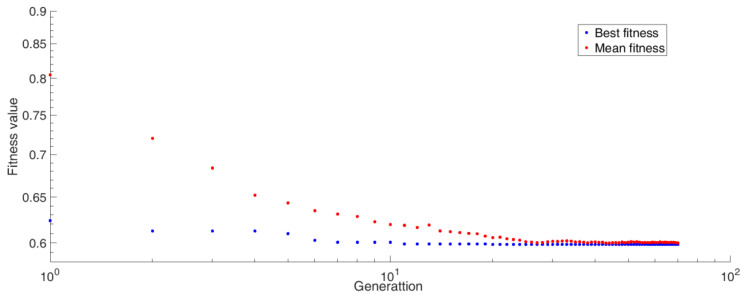
The convergence process of fitness function value.

In the optimization design process, two kinds of weight factors were used: $a_{1}\;$= 0.2, $a_{2}$ = 0.8 and $a_{1}$ = 0.8, $a_{2}$ = 0.2. According to Equation (1), the low density of the material was the main focus in the optimization based on the former weight factors, while the good shielding performance of the material was more the main focus in the optimization based on the latter. For the latter, the convergence process of fitness function value is shown in Figure 6. The final optimization results of the material parameters for different weight factors are shown in Table 4.

As can be seen from Table 4, when the optimization process focused on the material density, i.e., $a_{2}$ = 0.8, the mass fraction of B_4_C was larger, and the density of the material was smaller. When the shielding performance was the main focus, that is, $a_{1}$ = 0.8, the total dose equivalent behind the material for one fission was smaller. At the same time, in comparison with materials with different content and size of B_4_C, the designed materials showed better shielding performance, and this indicates that the optimized design method was effective. In general, the shielding performance of the two optimized materials was similar, mainly because there was little difference in the content of B_4_C in the two materials, and the difference caused by the particle size of B_4_C for fission neutrons was also small [22]. Obviously, a lower density of the material could be achieved by increasing the B_4_C content.

### 4.2. Neutron Transmission Experiment Results

**Figure 7:**
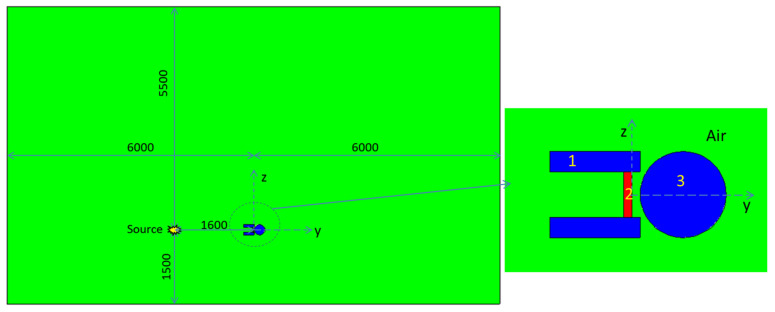
Neutron transmission simulation model. 1: Polyethylene shielding layer; 2: material sample (BCC Model for DM and Uniform Filling Model for BSS); 3: neutron ambient dose equivalent (rate) meters; 4: the green color indicates the surrounding air. The dimension of the x-axis is consistent with that of the y-axis, that is 6000 mm.

**Figure 8:**
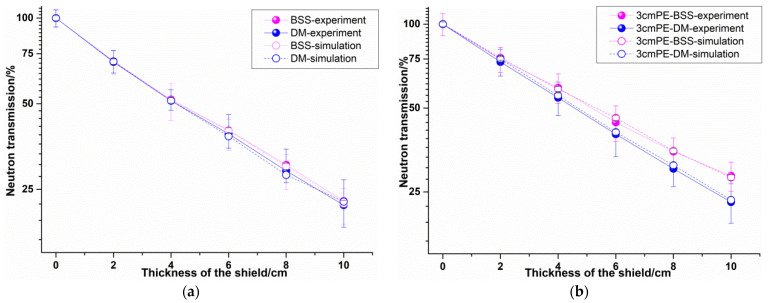
Neutron transmission experiment and simulation results. (**a**) Results of the condition without a polyethylene moderating layer, (**b**) results of the condition with a 3 cm polyethylene moderating layer.

Due to the 4 π angular emission of the neutron source, the scattering effect was great in the experiment. Therefore, according to the experimental layout, MCNP simulation was also carried out, which was consistent with the experimental results. In the modeling process, the experimental setting was maintained, including the distance between source and detector, that between detector and material, etc. The surrounding air, ground, sample table, and other structures were also considered, as shown in Figure 7. The experiment and simulation results are shown in Figure 8.

Generally speaking, the regularity of simulation and experimental results is consistent in Figure 8, that is, the neutron shielding performance of the designed material (DM) is comparable to that of the BSS material.

However, we found little difference in the neutron shielding performance between the designed material and the BSS material in the absence of a polyethylene moderating layer. When there was a 3 cm polyethylene moderating layer, the difference in shielding performance between the designed material and the BSS material became larger. That is, when the average neutron energy was 4.5 MeV, the neutron shielding performance of the designed material was equivalent to that of the BBS material and when the average neutron energy was 3.98 MeV, the neutron shielding performance of the designed material was slightly better than that of the BSS material. Because the proportion of intermediate and thermal neutrons in the neutron spectrum increased, as shown in Figure 3, the elastic scattering of B_4_C and the capture absorption of B element played an important role. Similarly, as the thickness of the material increased, the difference in neutron shielding performance between the designed material and the BSS material increased. This is also because the proportion of intermediate and thermal neutrons in the neutron energy spectrum increased with the increase of the material thickness. The moderating polyethylene reduced the flux of neutrons, so the measurement error became larger after adding a 3 cm polyethylene moderating layer. However, combined with the simulation results, it can be seen that the experimental results were still valid.

### 4.3. γ Rays Transmission Experiment Results

**Figure 9:**
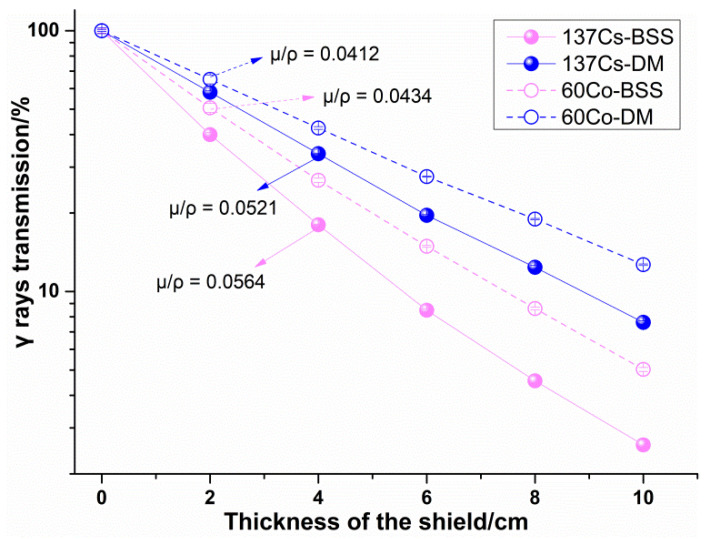
γ rays transmission experiment results.

The γ rays transmission of the two kinds of materials was also obtained by Equation (7), as shown in Figure 9. In evaluating the γ ray shielding property of materials, linear attenuation coefficient and mass attenuation coefficient are two key parameters. Therefore, the data were processed according to Equation (1) to obtain these two parameters of the two materials, as shown in Table 5.

It can be seen from Figure 8 that the γ rays transmission of the designed material was much higher than that of BBS, that is, the γ ray shielding performance of BSS was better than that of the designed material. In addition, the linear attenuation coefficient of the designed material was about 34.9% lower than that of the BBS material for ^60^Co and about 36.6% lower for ^137^Cs. However, since the density of the designed material was 5.21 $g\cdot {\mathrm{cm}}^{-3}$, the mass attenuation coefficient of the designed material was equivalent to that of BBS, which is about 5.07% and 7.62% less for ^60^Co and for ^137^Cs, respectively.

## 5. Conclusions

A high-boron-content composite material for neutron and γ rays shielding was developed, which consists of spherical B_4_C particles with a radius of 1.53 mm as the functional particles and 304 stainless steel as the matrix. Its features are summarized as follows.

(a) The B_4_C content of the newly designed material is 24.68 wt%, and the B content is nearly 20 wt%. The density of the prepared material sample was 5.21 $g\cdot {\mathrm{cm}}^{-3}$, much lower than that of borated stainless steel containing 1.7 wt% of boron (BSS_1.7_).

(b) The neutron shielding performance of the designed material appeared to be equivalent to that of BSS_1.7_ when the average neutron energy was 4.5 MeV; the neutron shielding performance of the designed material was better when the average neutron energy was 3.98 MeV.

(c) For γ rays, the linear attenuation coefficient of the designed material was lower than that of BSS_1.7_. However, the mass attenuation coefficient of the designed material was equivalent to that of BSS_1.7_; which were about 5.07% and 7.62% less than that of BSS_1.7_ for ^60^Co and ^137^Cs, respectively.

It can be concluded that the design method could be applied to other radiation shielding materials, and this designed material could be a candidate material for nuclear power plants or spent-fuel storage and transportation containers with high requirements for mobility. There is no denying that the mechanical and thermal properties of the new material should also be verified experimentally for its practical application.

## Figures and Tables

**Table 1:** γ ray linear attenuation coefficients (${\mathrm{cm}}^{-1}$ ) of the main element.

Energy (MeV)	Element		
	Fe	C	B
1 × 10^−3^	7.150 × 10^4^	3.980 × 10^3^	2.864 × 10^3^
5 × 10^−3^	1.100 × 10^3^	3.442 × 10^1^	2.256 × 10^1^
1 × 10^−2^	1.343 × 10^3^	4.271	2.924
0.5	6.622 × 10^−1^	1.569 × 10^−1^	1.879 × 10^−1^
2.0	3.357 × 10^−1^	7.996 × 10^−2^	9.572 × 10^−2^
10	2.356 × 10^−1^	3.526 × 10^−2^	4.089 × 10^−2^

**Table 2:** Neutron macroscopic cross section (${\mathrm{cm}}^{-1}$ ) of the main nuclide.

Energy (MeV)	Nuclide		
	^56^Fe	^12^C	^10^B
2.53 × 10^−8^	1.35	0.45	539.84
5 × 10^−5^	0.97	0.43	12.40
5 × 10^−2^	0.33	0.41	0.72
Fission (average: 1.98)	0.23	0.15	0.31
2.45	0.28	0.14	0.29
14.1	0.22	0.12	0.22

**Table 3:** Parameters of the γ rays source used in the experiments.

Source	Energy/MeV	Source Activity/Bq
^60^Co	1.25 (Average)	1.38 × 10^13^
^137^Cs	0.662	2.73 × 10^12^

**Table 4:** Parameters of the designed materials for different weight factors and results of the compared materials.

Weight Factors	Optimization Parameters			
	B_4_C Mass Fraction/%	Radius/mm	Density $/g\cdot {cm}^{-3}$	Total Dose Equivalent Behind the Shield for One Fission/Sv
$a_{1}$$=0.2,\;a_{2}$ = 0.8	24.68	1.53	5.17	1.05 × 10^–13^
$a_{1}$$=0.8,\;a_{2}$ = 0.2	23.97	1.728	5.23	1.01 × 10^–13^
Compared materials	1	1.53	7.72	1.76 × 10^–13^
	5	1.53	7.12	1.49 × 10^–13^
	10	1.53	6.50	1.34 × 10^–13^
	20	1.53	5.53	1.11 × 10^–13^
	24.68	1.00	5.17	1.05 × 10^–13^
		2.00	5.17	1.06 × 10^–13^
	25	1.53	5.14	1.10 × 10^–13^

**Table 5:** Linear attenuation coefficient and mass attenuation coefficient of the materials.

Material		BSS	DM
Density/$g\cdot {\mathrm{cm}}^{-3}$		7.6	5.21
Linear attenuation coefficient/${\mathrm{cm}}^{-1}$	^60^Co	0.3300	0.2147
	^137^Cs	0.4283	0.2716
Mass attenuation coefficient/${\mathrm{cm}}^{2}\cdot g^{-1}$	^60^Co	0.0434	0.0412
	^137^Cs	0.0564	0.0521

## Data Availability

Not applicable.
